# Use of Antibiotic-Eluting Calcium Sulfate in the Management of Post-surgical Spinal Infections and Spondylodiscitis: A Case Series

**DOI:** 10.7759/cureus.112081

**Published:** 2026-07-05

**Authors:** Bharatkumar R Dave, Ajay Krishnan, Shivanand C Mayi, Ravi Ranjan Rai, Mirant B Dave, Mikeson Panthackel, Rushikesh B Shahade, Arjit Vashishtha, Saurabh S Kulkarni, Yogenkumar Adodariya, Kishan Panjwani

**Affiliations:** 1 Spine Surgery, Stavya Spine Hospital and Research Institute, Ahmedabad, IND; 2 Spine, Bhavnagar Institute of Medical Science (BIMS), Bhavnagar, IND; 3 Spine, Stavya Spine Hospital and Research Institute, Ahmedabad, IND; 4 Orthopedics, King Edward Memorial Hospital and Seth Gordhandas Sunderdas (GS) Medical College, Mumbai, IND; 5 Orthopedics, Mahatma Gandhi Medical College and Research Institute, Aurangabad, IND

**Keywords:** antibiotic coated beads, postoperative wound infection, spine infection, spondylodiscitis, stimulan, surgical site infection (ssi)

## Abstract

Postoperative spinal infections, including spondylodiscitis and surgical site infections (SSI) following instrumentation, remain significant complications associated with considerable morbidity and complex management. Standard treatment often involves extensive surgical debridement, implant removal or revision, and prolonged systemic antibiotic therapy; however, achieving adequate local antibiotic concentrations can be challenging. In this retrospective case series, we evaluate the use of bioabsorbable calcium sulfate as a local antibiotic delivery system in eight adult patients (≥18 years; four women; mean age: 58.6 years) managed for post-instrumentation spinal infections between 2021 and 2025. Patient inclusion required a confirmed deep surgical site infection or spondylodiscitis with implant involvement, while patients with severe renal impairment or superficial infections were excluded. Surgical management included thorough debridement combined with implant removal or revision and, where indicated, reconstructive procedures such as corpectomy and interbody fusion. Antibiotic-loaded calcium sulfate (Stimulan, Biocomposites Ltd., Staffordshire, United Kingdom) beads were used for local antibiotic delivery based on intraoperative assessment and microbiological considerations. All patients tolerated the procedure well, with no immediate complications attributable to the calcium sulfate implantation. Favorable clinical outcomes were observed in all cases, with the resolution of symptoms, normalization of inflammatory markers, and radiological evidence of infection control during follow-up. The mean duration of hospital stay was 6.0 days. These findings suggest that antibiotic-eluting calcium sulfate is a safe and effective adjunct in the management of postoperative spinal infections, facilitating targeted local antibiotic delivery and contributing to successful infection control.

## Introduction

Recent advances in local antibiotic delivery systems have expanded the therapeutic options for managing complex spinal infections. Among these, antibiotic-loaded calcium sulfate has shown promising results in both spinal and orthopedic settings. Tang et al. demonstrated favorable clinical outcomes using antibiotic-loaded calcium sulfate beads in patients with spondylodiscitis, highlighting effective infection control and good clinical recovery [1]. Similarly, Bellini et al. reported encouraging preliminary results with the percutaneous use of absorbable antibiotic carriers in pyogenic spondylodiscitis, emphasizing its feasibility and safety [2]. In addition, Viswanathan et al. described the successful management of lumbosacral surgical site infections using antibiotic-impregnated beads, with significant clinical improvement and infection resolution [3]. A larger clinical series by Shah et al. further supported the effectiveness of dissolvable calcium sulfate carriers for local antibiotic delivery in orthopedic infections, demonstrating favorable outcomes in a substantial patient cohort [4].

Despite these encouraging findings, postoperative spinal infections remain a significant clinical challenge. Spinal instrumentation and fusion procedures are associated with a variable yet clinically significant risk of surgical site infection (SSI), with reported incidence ranging from 0.7% to over 10% in high-risk patient populations [5]. Deep infections involving the intervertebral disc, vertebral bodies, or implanted hardware, commonly presenting as spondylodiscitis, are particularly difficult to manage and are associated with increased morbidity, prolonged hospitalization, and the potential need for multiple surgical interventions [6].

The management of such infections typically requires a multidisciplinary approach, incorporating aggressive surgical debridement, implant retention or removal depending on mechanical stability and infection severity, and prolonged systemic antimicrobial therapy guided by microbiological findings. However, systemic antibiotic therapy alone is often insufficient due to poor vascularity at the infected site and the presence of bacterial biofilms on implants, which significantly impair antibiotic penetration and contribute to persistent infection [7,8]. Achieving high local antibiotic concentrations at the site of infection while minimizing systemic toxicity is therefore critical for successful treatment. Bioabsorbable carriers such as calcium sulfate (Stimulan, Biocomposites Ltd., Staffordshire, United Kingdom) provide an effective solution by enabling localized, high-dose antibiotic delivery with predictable elution kinetics, complete resorption, and the additional potential benefit of osteoconductive properties.

In this context, the present case series aims to evaluate the clinical utility, safety profile, and early outcomes associated with the use of antibiotic-eluting calcium sulfate as an adjunct to surgical debridement and implant management in patients with post-surgical spinal infections and spondylodiscitis.

## Materials and methods

Study design and patient selection

This retrospective case series was conducted at Stavya Spine Hospital and Research Institute, Ahmedabad, a tertiary referral spine center in India, and included eight patients who underwent surgical management for postoperative spinal infections following instrumentation between 2021 and 2025. Approval from the Stavya Spine Hospital and Research Institute Institutional Ethics Committee (IEC) was obtained prior to the commencement of the study (approval number: SSHRI/CS/NS/Stimulan/BRD/116/04.26). The study was conducted in accordance with the ethical principles outlined in the Declaration of Helsinki. Inclusion criteria were patients aged 18 years or older with a confirmed diagnosis of postoperative spinal infection, including spondylodiscitis or deep surgical site infection (SSI) with implant involvement, who underwent surgical intervention with the intraoperative use of 10 cc of calcium sulfate (Stimulan Rapid Cure, Biocomposites Ltd., Staffordshire, United Kingdom) as a local antibiotic delivery system. Patients with severe renal impairment, known antibiotic hypersensitivity, or inadequate soft tissue coverage were excluded. High-risk patients with comorbidities, including diabetes or urinary tract infections, were included, given the potential prophylactic and therapeutic benefit of local antibiotic delivery. Exclusion criteria included patients with superficial wound infections without deep involvement, those with active systemic infections unrelated to the spinal procedure, or patients with known allergies to any of the antibiotics used in the calcium sulfate carrier. The selection of antibiotics incorporated into the calcium sulfate carrier was individualized based on intraoperative findings and, where available, microbiological culture and sensitivity results. Antibiotics used in Stimulan were selected based on intraoperative culture results, local antibiograms, and pathogen susceptibility patterns. Vancomycin, gentamicin, amikacin, and streptomycin were most commonly used. No resistance was observed in this cohort, although a systematic evaluation of resistance emergence was not performed.

Surgical technique and treatment protocol

All procedures were performed under general anesthesia. The infected site was approached through the previous surgical incision whenever feasible; however, the extension of the incision or a new surgical approach was utilized when required to achieve adequate exposure. A standardized surgical protocol was followed in all cases.

Implant Management

Total or partial implant removal or exchange was performed in most patients to facilitate adequate debridement and eliminate colonized hardware. In cases where spinal stability was compromised, revision instrumentation and stabilization procedures were carried out as indicated.

Surgical Debridement

The extensive and meticulous debridement of all infected and necrotic tissues, including disc material and the involved bone, was performed. Debridement was continued until healthy, viable bleeding tissue was identified, ensuring adequate clearance of the infectious focus.

Local Antibiotic Delivery

Following thorough irrigation and final wound lavage, antibiotic-loaded calcium sulfate (Stimulan) was prepared intraoperatively by mixing with broad-spectrum antibiotics, commonly vancomycin, gentamicin, amikacin, or their combinations. The prepared material was then placed into the surgical site, including dead space, interbody regions, or around retained or revised instrumentation, to achieve high local antibiotic concentrations.

Wound Closure

The wound was closed in layers over a suction drain to prevent postoperative fluid accumulation and to allow the monitoring of drainage.

Systemic Antibiotic Therapy

Postoperatively, all patients received systemic intravenous antibiotics tailored according to microbiological culture and sensitivity results when available or empirical broad-spectrum therapy as guided by the infectious disease team. Antibiotic therapy was continued for an appropriate duration to ensure the complete resolution of infection.

Outcome measures

Clinical, laboratory, and radiological outcomes were systematically assessed in all patients. The resolution of symptoms such as lower back pain, radicular pain, and sinus discharge was also documented. Laboratory parameters, including erythrocyte sedimentation rate (ESR), C-reactive protein (CRP), and white blood cell (WBC) counts, were measured preoperatively and during follow-up to monitor systemic inflammation. Radiological outcomes were assessed using postoperative X-rays and MRI/CT scans to evaluate infection resolution, bone healing, and the integration of any grafts or instrumentation.

Statistical analysis

Continuous variables, including age, the duration of symptoms, and the length of hospital stay, were reported as mean ± standard deviation and range. Categorical variables, such as gender, comorbidities, infection site, and the type of surgery, were expressed as counts and percentages. Due to the small sample size, no inferential statistical analyses were performed. All data were analyzed using Microsoft Excel 2021 (Microsoft Corp., Redmond, WA) for descriptive statistics.

## Results

**Figure 1:**
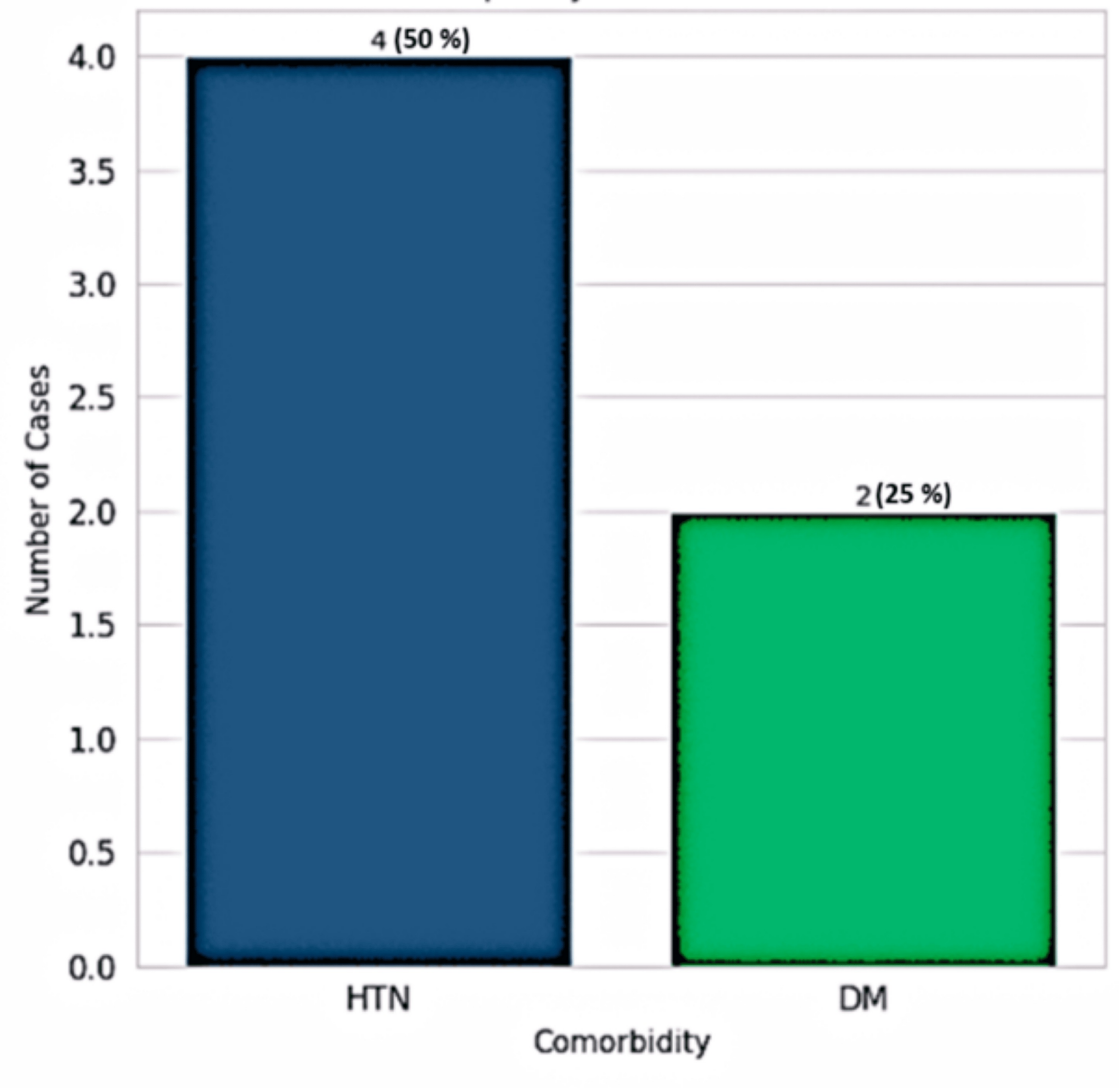
Distribution of Comorbidities Among Patients With Postoperative Spinal Infections Distribution of comorbid conditions, with hypertension (HTN) and diabetes mellitus (DM) being the most common

Eight patients (four women and four men) were included in this case series, with a mean age of 58.6 years (range: 18-79 years). As summarized in Table 1, the most frequently affected spinal region was the lumbosacral spine, while comorbidities such as hypertension (n = 5) and diabetes mellitus (n = 3) were prevalent among the cohort, as depicted in Figure 1. A majority of patients presented with spondylodiscitis or surgical site infection with implant involvement, demonstrating elevated inflammatory markers preoperatively. Figure 1 illustrates the distribution of comorbidities within the patient population. Collectively, these integrated clinical and demographic features highlight the relevance of careful patient selection and vigilant monitoring when utilizing antibiotic-loaded calcium sulfate in managing postoperative spinal infection.

**Table 1:** Summary of Clinical Characteristics, Surgical Management, and Outcomes of Spinal Infection Cases Treated With Antibiotic-Loaded Calcium Sulfate (Stimulan) LBP, lower back pain; RLL, right lower limb; LLL, left lower limb; BLL, bilateral lower limbs; SSI, surgical site infection; TLIF, transforaminal lumbar interbody fusion; BMP, bone morphogenetic protein; F, female; M, male

Case	Age (years)/sex	Complaint	Duration since symptoms	Site/diagnosis	Treatment	Antibiotic used in Stimulan	Length of hospital stay (days)	Systemic antibiotics	Final follow-up
1	64/F	LBP with RLL pain	1 year	L4-L5 spondylodiscitis	Implant removal and Stimulan	Amikacin + vancomycin	3	Amikacin + ceftriaxone and sulbactam + clindamycin (oral)	No pain
2	59/F	LBP	2 months	L4-L5 SSI	Implant removal, debridement, and Stimulan	Vancomycin + gentamicin	6	Amikacin + vancomycin + ceftriaxone and sulbactam + linezolid (oral)	Complete pain relief
3	79/F	LBP with LLL pain	2 months	L4-L5 implant-associated vertebral osteomyelitis	TLIF L3-L5; Stimulan	Amikacin + gentamicin	4	Amikacin + ceftriaxone and sulbactam + clindamycin (oral)	Complete remission of pain
4	76/M	LBP with LLL pain	2 months	L1-L2 spondylodiscitis	Anterior reconstruction of cage and Stimulan + BMP	Amikacin	7	Amikacin + ceftazidime and avibactam	Complete remission of pain
5	27/F	LBP with LLL pain	2 months	L5-S1 spondylodiscitis	Implant removal, revision instrumentation, and Stimulan	Amikacin	6	Meropenem + minocycline	Complete resolution of symptoms
6	61/M	LBP with BLL pain	6 months	L4-S1 spondylodiscitis and implant loosening	Implant removal, debridement, instrumentation, and Stimulan	Amikacin	10	Amoxicillin and clavulanic acid (oral)	Pain relief
7	59/M	LBP with BLL pain	4.5 months	D12-L1 SSI; D7-D8 spondylodiscitis	D7-D8 TLIF; D12-L1 transforaminal Stimulan injection	Amikacin	7	Amikacin + meropenem + minocycline (oral)	Symptom resolution
8	18/M	LBP	7 months	L1-L2 and L4-L5 SSI (chronic discharging sinus with implant loosening)	Implant removal and Stimulan	Vancomycin + streptomycin	5	Amikacin + ceftriaxone + teicoplanin + minocycline	Radiological confirmation of complete healing

**Figure 2:**
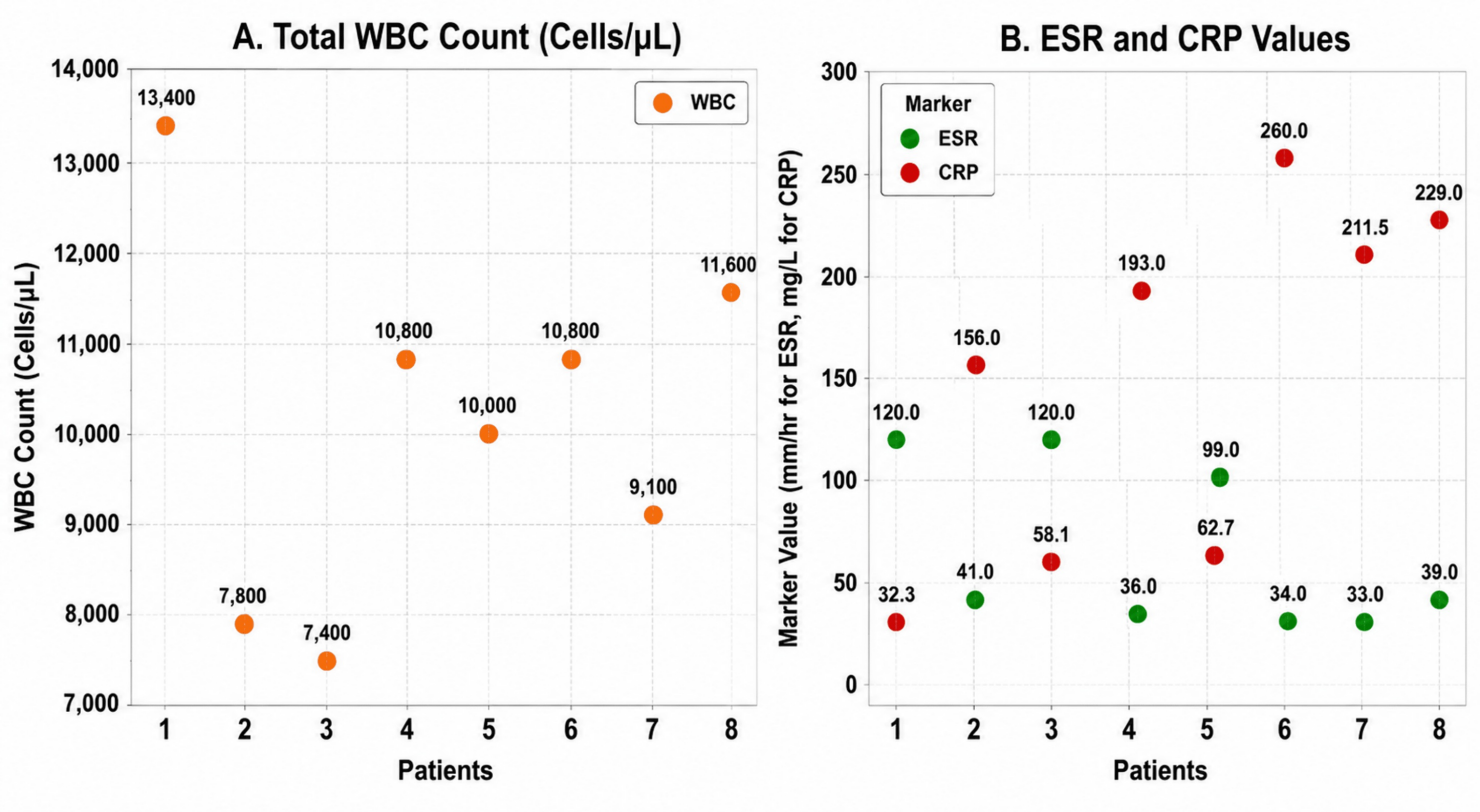
Preoperative Inflammatory Markers in Patients With Postoperative Spinal Infections Erythrocyte sedimentation rate (ESR) (reference range: 0-20 mm/hour), C-reactive protein (CRP) (reference range: <10mg/L), and/or total leukocyte count (reference range: 4000-11000 cells/μL), consistent with active infection WBC: white blood cell

The lumbosacral spine was the most frequently involved region (n = 7), with a single case involving the thoracic spine. Based on clinical and radiological evaluation, the infections were categorized as spondylodiscitis (n = 4), postoperative surgical site infection (SSI) (n = 2), and chronic discharging sinus with implant loosening (n = 2). All patients demonstrated biochemical evidence of active infection at presentation, with elevated preoperative inflammatory markers, including erythrocyte sedimentation rate (ESR), C-reactive protein (CRP), and/or total leukocyte count, as depicted in Figure 2.

All patients underwent surgical intervention as per the planned protocol, including thorough debridement with implant removal or revision as indicated, along with local antibiotic delivery using calcium sulfate. The application of Stimulan varied according to the intraoperative findings, including placement within debrided cavities, interbody spaces, or around retained/revised instrumentation.

**Figure 3:**
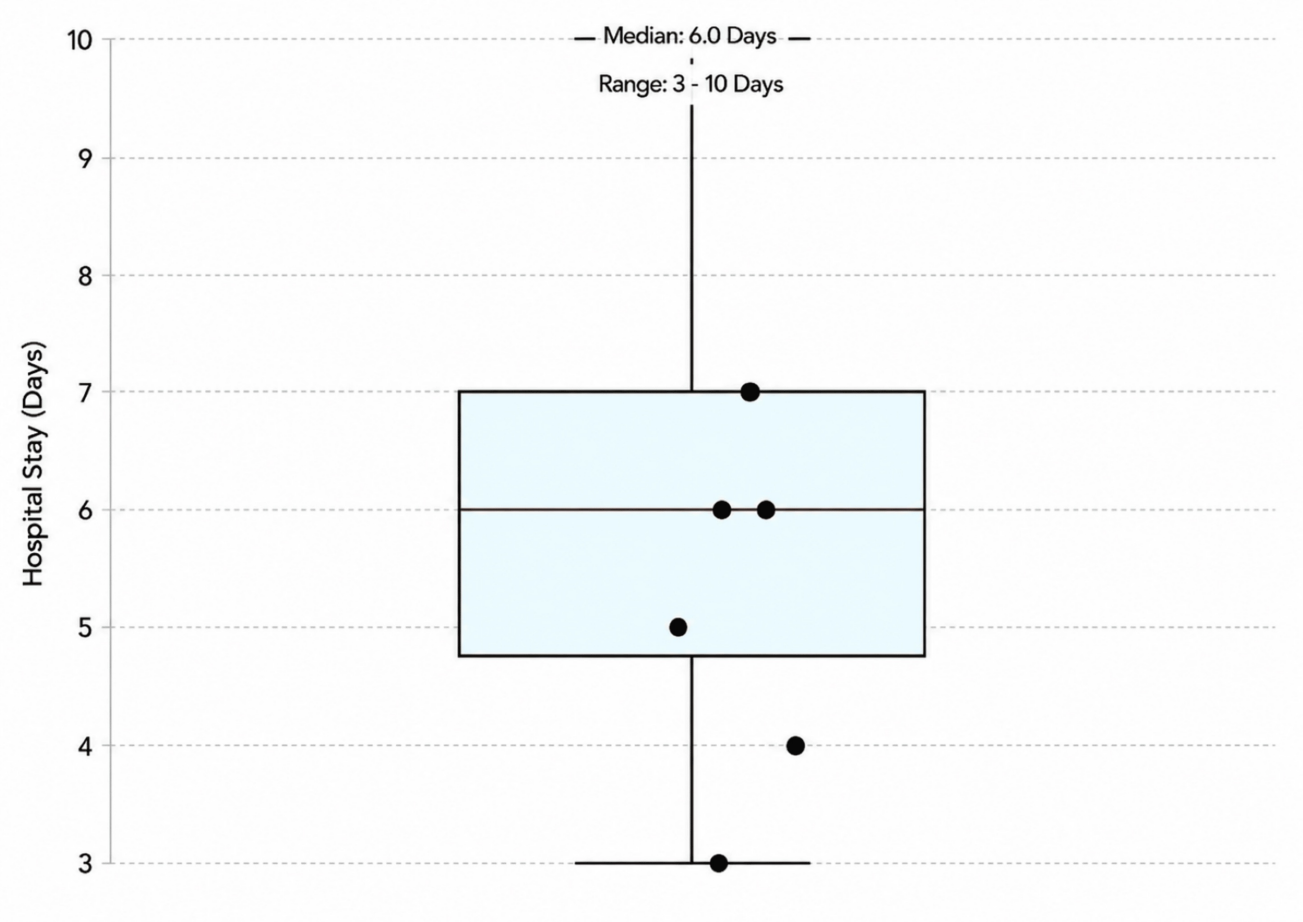
Length of Hospital Stay Among Patients Treated With Antibiotic-Loaded Calcium Sulfate Duration of hospital stay in days (mean, 6; range, 3-10)

The mean duration of hospital stay was 6.0 days (range: 3-10 days) (Figure 3). No intraoperative or immediate postoperative complications attributable to the use of calcium sulfate were observed, including the absence of local tissue toxicity, allergic reactions, or sterile wound-related complications during the hospital stay. Although no complications occurred in this cohort, known risks include persistent wound drainage, local inflammation, or transient hypercalcemia. Management would involve wound care, drain adjustments, anti-inflammatory therapy, monitoring serum calcium, and, rarely, surgical irrigation or removal if needed.

All patients received adjunctive systemic antibiotic therapy based on microbiological culture results and infectious disease recommendations. Antibiotics were given for a period of three months (six weeks of intravenous, followed by six weeks of oral dosing). At final follow-up, all patients demonstrated the clinical and biochemical resolution of infection, with the normalization of inflammatory markers. Symptomatic improvement was noted in all cases, with the complete resolution of pain or significant pain reduction and improvement in functional status. No cases of persistent infection, recurrence, or need for revision surgery were observed during the follow-up period. The follow-up period ranged from three to 12 months. No recurrence of infection was observed during this period, and radiological imaging confirmed bone integration and the resolution of spondylodiscitis or SSI in all cases.

**Figure 4:**
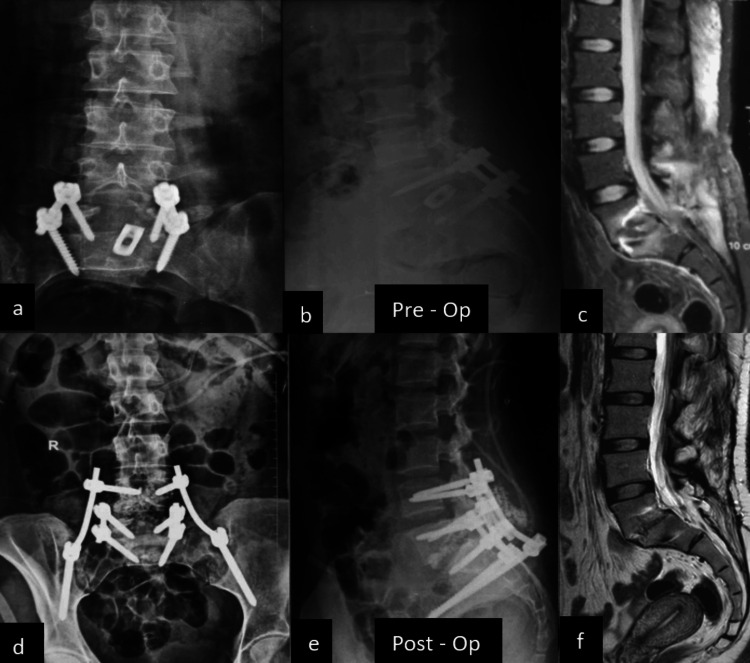
Preoperative and Postoperative Radiological Images of a Patient With L5-S1 Spondylodiscitis With Implant In Situ Operated With Cage Removal and Revision Implantation, Increasing Proximal and Distal Anchorage With Stimulan Insertion (Case 5) (a) Preoperative AP LS spine X-ray; (b) preoperative lateral LS spine X-ray; (c) preoperative sagittal T2W MRI image; (d) immediate postoperative AP LS spine X-ray; (e) immediate postoperative lateral LS spine X-ray; (f) postoperative sagittal T2W MRI image at six months T2W, T2-weighted; LS, lumbosacral

Figure 4 shows a case with preoperative and postoperative radiological images of a patient with L5-S1 spondylodiscitis with an implant in situ operated with cage removal and revision implantation, increasing proximal and distal anchorage with Stimulan insertion.

## Discussion

Postoperative spinal infections, including spondylodiscitis and deep surgical site infections (SSI), represent serious complications following spinal instrumentation, contributing significantly to patient morbidity, prolonged hospitalization, and increased healthcare costs [5]. The management of these infections is particularly challenging due to the presence of spinal implants, which act as a nidus for bacterial colonization and biofilm formation. Biofilms confer resistance to host immune mechanisms and significantly reduce the efficacy of systemic antibiotics, thereby necessitating combined surgical and adjunctive therapeutic strategies [6].

In the present case series, we evaluated the role of antibiotic-eluting calcium sulfate as an adjunct to surgical debridement and implant management in eight patients with postoperative spinal infections. Our results demonstrate consistent and favorable outcomes, with the complete clinical, biochemical, and radiological resolution of infection in all cases and no recurrence during follow-up. These findings highlight the potential of combining aggressive surgical debridement with targeted local antibiotic delivery to optimize infection control in complex spinal infections [9].

The cornerstone of management in such cases remains adequate surgical debridement, which reduces bacterial load, removes necrotic tissue, and disrupts biofilm architecture [7]. In our series, all patients underwent extensive debridement, along with implant removal or revision where indicated, in line with established treatment principles [7]. However, systemic antibiotic therapy alone is often inadequate due to poor vascularity at the infection site and the presence of biofilm-associated microorganisms, which significantly limit antibiotic penetration [6,8].

Local antibiotic delivery systems have therefore emerged as a valuable adjunct, allowing the administration of high concentrations of antibiotics directly at the site of infection. Calcium sulfate-based carriers, such as Stimulan, offer distinct advantages over traditional polymethyl methacrylate (PMMA) beads. These include complete bioresorbability, the elimination of the need for secondary removal surgery, and predictable antibiotic elution kinetics [10]. Importantly, local antibiotic concentrations achieved with calcium sulfate have been shown to exceed minimum inhibitory concentrations (MIC) for common pathogens, including resistant organisms, without increasing systemic toxicity [9].

The clinical efficacy of antibiotic-loaded calcium sulfate in spinal infections is increasingly supported by contemporary literature. Tang et al. reported favorable outcomes in patients with spondylodiscitis treated with calcium sulfate beads, demonstrating effective infection control and improved functional outcomes [1]. Bellini et al. further demonstrated the feasibility of the percutaneous application of absorbable antibiotic carriers in pyogenic spondylodiscitis, highlighting its role in reducing surgical morbidity while maintaining efficacy [2]. Viswanathan et al. reported the successful management of lumbosacral SSI using antibiotic-impregnated beads, with significant clinical and laboratory improvement [3]. Similarly, Shah et al. reported favorable outcomes in a large cohort of orthopedic infections treated with dissolvable calcium sulfate, reinforcing its safety and effectiveness as a local antibiotic delivery system [4]. Our findings are consistent with these reports and further extend their applicability to complex post-instrumentation spinal infections.

In addition to its role in antibiotic delivery, calcium sulfate may provide osteoconductive properties that facilitate bone regeneration and structural support, particularly in cases requiring interbody fusion or anterior column reconstruction [11]. This is particularly relevant in spinal infections, where both the eradication of infection and the restoration of spinal stability are critical for optimal outcomes. In our series, calcium sulfate was successfully utilized in conjunction with reconstructive procedures, without compromising clinical outcomes.

A key concern associated with calcium sulfate use is the potential for complications such as persistent wound drainage, local inflammatory reactions, and, rarely, hypercalcemia. Previous studies have reported variable rates of such complications, often related to excessive volume or the suboptimal placement of the material [12]. However, in our series, no complications attributable to calcium sulfate were observed, likely due to controlled dosing, appropriate case selection, and meticulous surgical technique. This underscores the importance of adherence to standardized protocols when utilizing such biomaterials.

The mean duration of hospital stay in our study was relatively short (six days), which may reflect effective early infection control and rapid postoperative recovery. Additionally, all patients demonstrated the normalization of inflammatory markers (ESR and CRP) and significant symptomatic improvement, particularly with respect to pain relief and functional status. These findings suggest that the combined approach of surgical debridement and local antibiotic delivery may contribute to faster clinical recovery and reduced healthcare burden. Importantly, no complications such as local tissue toxicity, allergic reactions, or persistent wound drainage were observed in this cohort [13-15]. Although previous studies have reported wound-related complications with calcium sulfate use, these are often associated with higher volumes or suboptimal placement techniques. Careful intraoperative application and appropriate dosing in our series likely contributed to the favorable safety profile observed.

Despite these encouraging findings, certain limitations must be acknowledged. The small sample size and retrospective design limit the generalizability of the results. Additionally, long-term follow-up data regarding infection recurrence, bone integration, and the complete resorption of calcium sulfate were not fully captured in this study. Although this series demonstrates a positive safety and efficacy profile for antibiotic-loaded calcium sulfate, it is important to consider how it compares to alternative local antibiotic delivery methods. Conventional options, such as polymethyl methacrylate (PMMA) beads, have been widely used and offer local antibiotic release but require additional procedures for removal and are not bioresorbable. Other bioabsorbable materials, such as collagen sponges, may not provide the same sustained antibiotic elution as calcium sulfate. The predictable absorption and dual role of calcium sulfate in both antibiotic delivery and potential osteoconduction may offer practical advantages in spinal infection management. These findings must be interpreted with caution. Rigorously designed prospective studies with larger, controlled cohorts and extended follow-up are necessary to validate these outcomes and establish standardized treatment protocols.

Nevertheless, the results of this case series suggest that antibiotic-loaded calcium sulfate is a safe and effective adjunct in the management of postoperative spinal infection and may be extrapolated to a larger population of infected cases in future clinical practice. Further prospective studies are warranted to optimize antibiotic combinations, dosing strategies, and indications for their use.

## Conclusions

Antibiotic-eluting calcium sulfate represents a valuable bioabsorbable adjunct in the management of chronic and recurrent postoperative spinal infections. When combined with thorough surgical debridement and appropriate implant management, its use in this case series was associated with a favorable safety profile, with no observed procedure-related adverse events.

The ability of Stimulan to deliver high local concentrations of antibiotics while eliminating the need for secondary removal procedures makes it an attractive option in complex spinal infections. Additionally, its potential role as a prophylactic adjunct in high-risk patients, particularly those with significant comorbidities or concurrent infections such as urinary tract infections, may further enhance infection prevention strategies.
